# Quarterly Report of the Proceedings of the Epidemiological Society

**Published:** 1855-12

**Authors:** 


### QUARTERLY REPORT

OF THE

### Proceedings of the Epidemiological Society.

---

### PAPERS READ AT ORDINARY MEETINGS.

DURING the past quarter the following papers have been read at the General (monthly) Meetings of this Society:—

On Monday, April 2nd. "On Typhoid Fever, and the Absence of Typhus, in the towns of Chatham and Rochester, during the year 1854." By Frederick James Brown, M.D., of Chatham.

"On the occurrence of Fever at Sible Hedingham, Essex, and at Cowbridge, South Wales." By William Camps, M.D.

Mr. Charles Hawkins, Mr. Pilcher, Dr. Snow, and Mr. George Glover took part in the discussion of these papers.

On Monday, May 7th. "Some Account of the Bowel Diseases prevalent in the Crimean Expedition, during the winter." By William Smart, M. D., Surgeon of H. M. S. *Diamond*, hospital ship, at Balaklava. Communicated and read by Dr. McWilliam.

Dr. Babington, Dr. Bryson, and Dr. McWilliam took part in the discussion of this paper.

On Monday, June 4th. "On the Communication of Cholera through the Medium of Water." By John Snow, M.D. This paper was discussed by Dr. Greenhow, Mr. Tucker, the Rev. Mr. Whitehead, and Mr. French.

### DONATIONS TO THE SOCIETY.

During the quarter the following donations to the Funds of the Society have been received:—

| | £ | s. | d. |
|---|---|---|---|
| Sir Charles Mansfield Clarke, Bart. (a zealous supporter of the Society) . . . | 10 | 10 | 0 |
| William Samuel Jones, Esq., of Chester-terrace, Regent's-park . . . | 5 | 5 | 0 |
| Dr. Wilbraham Falconer, of Bath . . | 1 | 1 | 0 |
| Mrs. Richard Hall, 5, Devonshire-place, Haverstock-hill . . . . | 5 | 5 | 0 |

Upwards of twenty volumes of valuable works on Epidemic Diseases have been presented to the Society by Dr. Howell, of 15, Upper Montague-street.

Russell on the Plague. 4to.
Statistical Report of the Diseases of the West Indies.
,, of Western Africa.
,, of St. Helena, Cape of Good Hope, and the Mauritius.
,, of the United Kingdom.
,, of the Mediterranean and British America.
,, of Ceylon and the Burmese Empire.
Haygarth on the Small-Pox. 2 vols.
Rogers's Essay on Epidemic Diseases.
Ratty on the Weather and the Diseases of Dublin.
Brockelsby on Military Hospitals and Camp Diseases.
Pringle on the Diseases of the Army.
Rouppe on the Diseases of Seamen.
Lind on Hot Climates.
Huxham on Fever.
Hodges on the Plague.
Faulkner and Tully on the Plague.
Inquiry into the Causes of Pestilence.
Bateman on the Diseases of London.
Prichard on Epidemic Fever.
Cholera Gazette and Pamphlet on Cholera.

Webster on Epidemic Diseases. 2 vols.
Balfour on Sol Lunar Influence.
Hancock on Pestilence.

Several other donations of books have also been received from Dr. Barton, of New Orleans, Dr. McDonnell of the Poor Law Commission for Ireland; Dr. Aicken; The General Board of Health; the Weekly Board of the Middlesex Hospital; Dr. Bretts; James Fernandez Clarke, Esq.; Dr. Franque, of Wiesbaden in Nassau; Thomas Hopley, Esq.; Dr. Waller Lewis; and the Editors of "The Veterinarian," "The Pharmaceutical Journal," and the "Assurance Magazine."

A valuable communication on Cholera in Germany, has been received from Dr. Varrentrapp, of Frankfort.

### NEW MEMBERS.

The following gentlemen have been elected members of the Society during the quarter.

*As Resident Members.*—George Glover, Esq., Queen's-square, Westminster; R. K. Pierce, Esq., Notting-hill; Donald Fraser, M.D., Oakley-square, Hampstead-road, and William Munk, M.D., of Finsbury-place.

*As Non-resident Member.*—Thomas Hopley, Esq., Eastbourne, Sussex.

### VACCINATION.

On Saturday, the 16th June, a deputation of the Society, accompanied by Sir Benjamin Hall, President of the Board of Health, had an interview with the Right Hon. Sir George Grey, Secretary of State for the Home Department, on the subject of a Bill for the extension of Vaccination.

### DEATHS OF MEMBERS.

The Society has had to deplore the death of Dr. Hector Gavin, one of its most enterprising and useful members. The calamitous accident that caused this melancholy event adds peculiar poignancy to the regret which the Society, in common with his friends and the public, must feel for the loss they have sustained.

Death has also recently deprived the Society of another member, Mr. Charles Cochrane, so well known for his enthusiastic zeal in the promotion of all that relates to sanitary improvement.

### PAPERS TO BE READ.

On Monday, July 2nd, a Paper "on the Epidemic of Small-pox in Jamaica, in 1852, with observations on the Protective Value of Vaccination in Hot Climates," will be read by Dr. Edward Cator Seaton.

